# Spontaneous Visual Imagery During Meditation for Creating Visual Art: An EEG and Brain Stimulation Case Study

**DOI:** 10.3389/fpsyg.2019.00210

**Published:** 2019-02-22

**Authors:** Caroline Di Bernardi Luft, Ioanna Zioga, Michael J. Banissy, Joydeep Bhattacharya

**Affiliations:** ^1^School of Chemical and Biological Sciences, Queen Mary, University of London, London, United Kingdom; ^2^Department of Psychology, Goldsmiths, University of London, London, United Kingdom

**Keywords:** visual arts, EEG, transcranial alternating current stimulation (tACS), gamma oscillations, meditation, spontaneous visual imagery, entrainment, alpha oscillations

## Abstract

Experienced meditators often report spontaneous visual imagery during deep meditation in the form of lights or other types of visual images. These experiences are usually interpreted as an “encounters with light” and gain mystical meaning. Contrary to the well-studied intentional and controlled visual imagery, spontaneous imagery is poorly understood, yet it plays an important role in creativity of visual artists. The neural correlates of such experiences are indeed hard to capture in laboratory settings. In this case study we aimed to investigate the neural correlates of spontaneous visual imagery in an artist who experiences strong visual imagery during meditation. She uses these images to create visual art. We recorded her EEG during seven meditation sessions in which she experienced visual imagery episodes (visions). To examine the functional role of the neural oscillations we also conducted three separate meditation sessions under different transcranial alternating current (tACS) brain stimulation: alpha (10 Hz), gamma (40 Hz) and sham. We observed a robust increase in occipital gamma power (30–70 Hz) during the deepest stage of meditation across all sessions. This gamma increase was consistent with the experience of spontaneous visual imagery: higher during visions compared to no visions. Alpha tACS was found to affect the contents of her visual imagery, making them sharper, shorter and causing more visions to occur; the artist reported that these sharp images were too detailed to be used in her art. Interestingly, gamma and sham stimulation had no impact on the visual imagery contents. Our findings raise the hypothesis that occipital gamma might be a neural marker of spontaneous visual imagery, which emerges in certain meditation practices of experienced meditators.

## Introduction

In common terms, imaginative and creative are often used interchangeably to describe ideas/objects or the individuals producing them. However, creativity is not necessarily the same as imagination (Singer, 2011), and the relationship between imagery and creative cognition is multilayered. Psychologically, imagination is a broad term representing our almost unique ability to transcend the current constraints of space, time, and causality leading to mental simulation of future, creating fictional, unusual worlds, and experiences (Taylor, 2011); in essence, imagination includes both creative and non-creative thoughts.

Ward (1994) has argued for structured imagination, referring to a nonrandom but structured approach of generating new ideas and concepts; here, imagination is constrained by existing knowledge and categories. For example, when participants are asked to imagine animals living on a distant planet, their responses are structured by the properties of the animals living on the planet earth. Although this type of imaginative thinking has been found to be quite useful in creative idea generation (Ward, 1995), it is nonetheless a targeted method of imagination which is devoid of spontaneity.

Whereas there is a role for spontaneous thoughts in the creativity research, especially in studies related to mind-wandering and creativity (Baird et al., 2012), the spontaneous imagery, on the other hand, is much less studied, and we know very little about the role of spontaneous imagery in creativity. Interestingly, spontaneous (visual) imagery is often associated with meditation. For example, in the well-known “encounters with light” experience, meditators, primarily practicing in the Buddhist tradition, report several forms of lights or luminous experiences (Lo et al., 2003; Lindahl et al., 2013). These visual images of inner light may be a special type of visual imagery, albeit a spontaneous one, which has been overlooked in the imagery literature as most studies on visual imagery have looked into voluntary visual imagery whose content rely heavily on working memory (Albers et al., 2013; Dentico et al., 2014). The close link between spontaneous imagery and meditation was speculated by Austin (2003) almost 40 years ago, “*The ease with which meditators can learn to let go and enter a satisfying state of calm, detached awareness does correlate with their basic ability to produce spontaneous visual imagery, to free associate, and to tolerate any unreal experiences that may occur*” (Austin, 2003, p. 184). However, the underlying neuronal correlates of these types of spontaneous visual imagery are not known and nor their potential links to creative cognition.

In the present study, we aimed to address these issues by adopting a phenomenological approach based on the first person experience (Lutz et al., 2002). Lia Chavez (L.C.) is a New York (United States) based professional artist who has featured in a number of internationally renowned venues. L.C. experiences intense visual imagery generated spontaneously during her meditations, and uses the content of these visual images experienced during her deep meditative state as a source of creative inspiration for her multimedia work. We performed multisession recording of EEG signals from L.C.’s brain during meditation and the experiences of visual imagery. Subsequently, we tested whether it was possible to modulate visual imagery experiences by administering transcranial brain stimulation, in particular, transcranial alternating current brain stimulation (tACS), during meditation. tACS is a noninvasive technique that can be used to modulate endogenous brain oscillations possibly through entrainment (Antal and Paulus, 2013). It has been successfully used to probe the functional role of certain oscillations (e.g., alpha and gamma) on perception and cognition (e.g., Laczo et al., 2012; Janik et al., 2015; Luft et al., 2018). This technique allowed us to explore the possibility of causally interfering with the visual imagery as experienced during meditation.

Therefore, our objectives were as follows: (1) to investigate oscillatory changes during different depth levels of meditation (three stages, see “Methods”); (2) to analyze the oscillatory correlates of each visual imagery episode during the deepest stage of meditation; (3) to explore the casual relationship of oscillatory changes during different levels by analyzing the effects of alpha, gamma and sham tACS, on her visual imagery during meditation. Based on prior neuroimaging work (e.g., Lehmann et al., 2001; Lutz et al., 2004; Cahn et al., 2010; Braboszcz et al., 2017), we predicted that occipital gamma would increase with the depth of meditation. Second, we predicted that during deep meditation, the increase in occipital gamma power would be higher during her visions rather than no-visions. Third, we predicted that gamma tACS would boost her visual imagery during deep meditation.

## Materials and Methods

**FIGURE 1:**
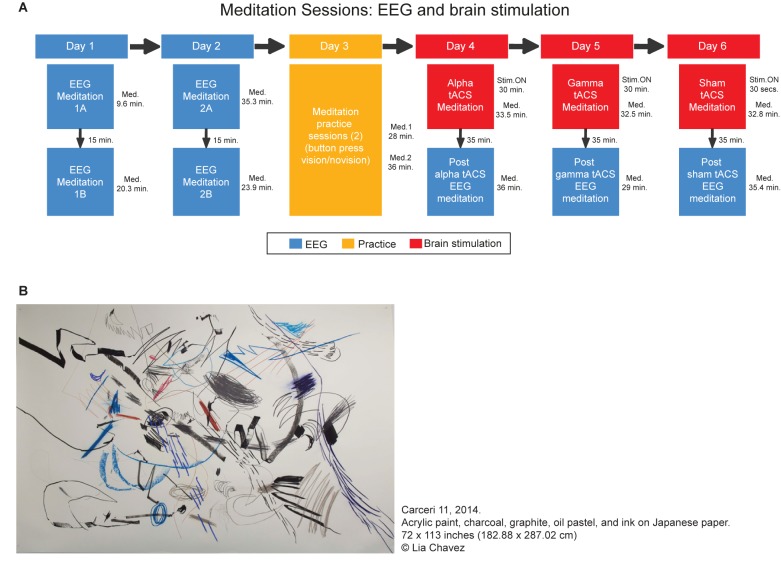
Overview of the meditation sessions. **(A)** The sessions in which the EEG was recorded are represented in blue whereas the meditation sessions that occurred under tACS are in red. A practice day is represented in yellow. In these practice sessions, the participant practiced the meditation indicating the onset and offset of the visions with a button press (once she was on stage 3). The duration of each session is presented next to each box. In the brain stimulation session, the duration of the stimulation is described as Stim ON whereas the total duration of the meditation in “Med.”. It is important to notice that in the tACS sessions the meditation occurred simultaneously with brain stimulation. In those cases, the tACS stopped after 30 min while the meditation stopped shortly after **(B)** Example of the art produced by Lia Chavez during her meditation process. Each individual element of the part represents a vision, which are drawn during her meditation, with eyes closed. *Carceri 11*, 2014. Acrylic paint, charcoal, graphite, oil pastel, and ink on Japanese paper. 72 × 113 inches (182.88 × 287.02 cm) Lia Chavez.

In this case study, a professional artist took part in 10 meditation sessions. These sessions took place over 6 separate days spread over a period of a few months. During seven sessions EEG (electroencephalogram) signals were recorded in order to investigate the large scale neural oscillatory changes during meditation. In the three other sessions, electrical brain stimulation (transcranial alternating current stimulation, tACS) was applied to modulate cortical activity in a frequency dependent manner in order to investigate the functional role of specific neural oscillations in meditation and associated creative imagery. The overview of the experimental sessions and a sample of her work developed based on her visual imagery are shown in Figure 1A,B. Written informed consent was obtained from the participant at each session. Further, our participant has accepted to have her identity disclosed in the paper. The experimental protocol was approved by the local Ethics Committee of the Department of Psychology at Goldsmiths, and all procedures were conducted in accordance with the Declaration of Helsinki.

### Participant

A New York-based internationally-exhibited artist, Lia Chavez (L.C. afterward), took part in this study. She is among Origin Magazine’s Top 100 Creatives Changing the World for 2015. Her work explores the phenomenology of light and the possibilities of using consciousness as an art material. She initially approached one of the authors (J.B.) detailing her experiences of spontaneous visual imagery during meditation as a source of her creative inspiration; subsequently, she expressed her willingness to participate in neuroimaging experiments to investigate the correlates between functional brain activities and “*the profound moments of interior visualizations … revealing the inception of the creative spark …”* (L.C., personal communication). L.C. has been practicing meditation intensely for over 10 years, including periods in which she meditated for lengthy periods of time (up to 10 h a day for 2 weeks at a time). Her meditation practice includes two different types of meditation according to Tibetan Buddhism: stabilizing and analytical (Chodron, 2007). Stabilizing can be considered as a strategy for quietening the mind by focusing the attention on simple repetition of words or mantras, on the breath, or even on a symbol within the mind. This type of meditation relies on serial repetition with the purpose to prepare the mind for a deeper kind of focused contemplation. The analytical meditation, on the other hand, is that state of deeper contemplation in which the meditator experiences a quiet mind in order to obtain a conceptual understanding of how things are, to a depth that would offer enough clarity and novel insight into the true nature of that concept. Both types of meditation can usually be combined within a single meditation session. L.C. reports using a variety of stabilizing techniques such as repeating a mantra, focusing on the pause between the in and out breath, and focusing attention on different body parts to generate heightened sensation. L.C. reports that once her mind is stable and a threshold is crossed, she goes into analytical contemplation which is the state that she experiences the spontaneous visions, which she calls “encounters with light.” During these spontaneous visions, she reports trying to remain detached from any emotions or judgments associated with the visual experience. This is how she describes her experience:

*“I integrate a variety of cross-disciplinary contemplative traditions into my artistic process as a way of exploring the inception of the creative spark, how the creative artist’s own ontology of becoming incarnates into art objects, and how this process might relate to the cosmological order. Durational analytic meditation is at the core of my process. As I’ve journeyed through deep meditation into the vast unknown of my own inner landscape, I’ve discovered that the silent mind is, in fact, the seeing eye within a great storm. In deep analytical meditation, I experience cataclysmic visions of vortices, fibers of electricity, clouds of short-lived photons, cascading firebolts, and embryonic stars. It’s a process which feels as though I am observing passionate and terrifying dances between the elements — a mental meteorology, if you will. As an artist who has always worked with light as a primary art material, you can imagine how powerful it was for me to encounter this experience for the first time in 2012. In time, I’ve come to discover that experiences of meditation-induced encounters with light is a widely-documented phenomenon throughout cross-cultural meditation traditions, most prominently within Buddhist meditation practice. Since first encountering these visions of luminous objects, I have cultivated durational analytic meditation as a source of inspiration for my visual and performance artwork.”* (L.C., personal communication). During her work, she depicts each vision on canvas with her eyes blindfolded. One example of the results can be visualized in Figure 1B. Her mixed media drawings are generated through several hours in a meditative state while continually blindfolded without sound. As she works, she positions herself atop the canvas surface and fashions complex gestural glyphs to depict her visions as they occur.

### Meditation Sessions

In each meditation session, the subject sat in her usual meditation posture on a flat chair holding a response box with four buttons. She was instructed to rest with her eyes closed for 5 min followed by another 5 min with eyes opened, both resting periods without any meditation. This allowed us to collect resting state neural recording and to make her feel familiarized and comfortable within the laboratory setting. Following the resting periods, she was instructed to start her meditation and she pressed the button 1 (stage 0) in the response box to indicate its onset. In stage 0, she was not yet meditating but attempting to do so. Once she started reaching what she considered as an initial meditative state, she pressed button 2 (stage 1). When the meditation advanced to a deeper stage, she pressed the button 3 (stage 2). Note that this stage 2 was associated with the transition from stabilizing to analytical meditation. As soon as she entered into deep state of analytical meditation, she pressed button 4 (stage 3), which is the stage in which she experiences her visions. It is important to notice that the meditative stages she indicated were based on her individual experience of meditation depth, which cannot be compared between people. She reported that her visions usually occur in her deepest meditative states only. What defined the meditation stage for her was the depth of the state and not the presence of visions. There was no time limit or any other constraint applied to our participant in relation to her meditation practice. Once stage 3 was finished, she pressed button 1 to signal the offset of the meditation session.

In days 6, the procedures for meditation were identical except that once she was on stage 3, she indicated the onset and offset of each vision. Both the onset and the offset of individual vision episode were registered by button press. Further, any change of the content of the vision, i.e., a vision was followed by another vision rather than a no vision, was also indicated by another button press. She had two sessions in a previous day (day 3) for practicing this technique but the EEG data for these two practice sessions were not analyzed. By using this procedure, we could quantify not only the duration of each vision but the number of different vision contents that occurred during the meditation. It is important to notice that all sessions were more than 24 h apart.

### EEG Recording and Analysis

Continuous EEG signals were recorded using 64 active electrodes using a BioSemi ActiveTwo amplifier. The electrodes were placed according to the extended 10–20 system of electrode placement. Vertical and horizontal electro-oculograms were recorded using four additional external channels to monitor eye movements. The signals sampled at 512 Hz, bandpass filtered between 0.16 and 100 Hz. The instruction screen, button presses and event timings were recorded using the MATLAB based toolbox Cogent 2000^1^. The EEG data was processed and analyzed by MATLAB based custom scripts and the following toolboxes: EEGLAB for preprocessing (Delorme and Makeig, 2004) and the signal processing toolbox in MATLAB. For preprocessing, we re-referenced the data to the arithmetic average of the two earlobes, and high-pass filtered at 0.5 Hz. The data was visually inspected for removal of visible artifacts such as muscle activity and eye-movements/saccades. For the vision vs. no-vision analysis, the data was also segmented into epochs of 2 s but the first epoch, immediately following the response, was excluded to avoid interference activity related to the button press.

In order to analyze the oscillatory response at each meditation stage, we estimated the power spectral density using the Welch’s method (averaged periodogram), by dividing the data into 2 s windows with an overlap of 50%. We estimated the spectral power from 1 to 80 Hz in steps of 0.5 Hz. The power values at each electrode and each condition were averaged based on the standard EEG frequency bands: delta (1–4 Hz), theta (4–8 Hz), alpha (8–12 Hz), beta (13–30 Hz), gamma 1 (30–45 Hz), and gamma 2 (55–80 Hz). For the vision vs. no vision comparison we selected broad gamma band from 30 to 80 Hz since there was no difference between gamma 1 and gamma 2. The EEG data was expressed as percentage changes from the baseline power at stage 0 (non-meditative state).

For statistical comparisons, we used the spectral power values for each epoch. In order to avoid circularity, the data of days 1 and 2 were used to guide the main comparison of the vision vs. no-vision analysis. The electrodes showing peak percentage increase (analysis merging all the sessions from day 1 and 2) were selected for the contrast between vision and no vision contrasts in the sessions following brain stimulation (days 4, 5, and 6). Since we only found a robust change in gamma band, we only compared visions vs. no visions in this frequency band.

### Brain Stimulation: tACS

Transcranial alternating current was delivered through a battery driven Neuroconn DC-Plus Stimulator. Two saline soaked sponged electrodes (5 × 5 cm = 25 cm^2^) were attached to conductive rubber electrodes attached to participants’ scalps with rubber head straps. A sinusoidal current of 1.5 mA peak-to-peak was applied at the frequency of 10 Hz for alpha tACS, 40 Hz for gamma tACS, and 10 Hz for sham (tACS duration was only for the first 30 s for sham) with a zero-degree phase offset and no DC offset. The electrodes were attached bilaterally on the occipital areas: electrodes PO7 and PO8 according to the extended 10–20 system. This montage was chosen for two reasons: (1) we needed to stimulate occipital areas as this was the area where we observed increased gamma oscillations in stage 3 of meditation; (2) we specifically chose PO7 and PO8 because this montage minimizes the risk of phosphenes as it reduces the current flow to the eyes (Laakso and Hirata, 2013). Additionally, we opted for traditional frequencies of stimulation (10 and 40 Hz) rather than individualized peak frequencies due to the difficulty of a clear peak in the gamma band. The stimulation lasted for 30 min and happened simultaneous to the meditation. After each session, the participant was asked to report any sensations she had experienced during the tACS session. She did not know the modality of the brain stimulation or the possibility of different frequencies. After the three sessions, she reported feeling a tickling sensation during the beginning, but she also reported that this sensation faded away shortly and she could not feel anything during the meditation (in all three sessions). After the last session, she was asked to indicate if she could guess whether the sessions were sham or stimulation. She reported having active stimulation during all sessions, which shows that she could not notice the difference between the sham and active stimulation sessions. This is expected since it was found that the cutaneous sensation persists after the stimulation is switched off on sham sessions which makes it hard for the participants to distinguish the stimulation conditions (Ambrus et al., 2012).

## Results

### Meditation EEG Sessions

As there was no constraint or interference with the meditation practice as performed by L.C. in the lab, the duration varied across sessions (see Figure 1). The first session was the shortest, with a total duration of 9.65 min (4.4 min on stage 1, 2.50 on stage 2, and 2.75 on stage 3). The longest session was the third (session 2A), which lasted for 35.30 min (1.10 min on stage 1, 11.50 on stage 2, and 22.70 on stage 3). The second session on the same day (session 2B) was also long during stage three (1.65 min on stage 1, 1.70 on stage 2, and 20.55 on stage 3). The second session of the first day (session 1B) was longer than the first but still much shorter than sessions 2A and B (1.2 min on stage 1, 8.90 on stage 2, and 10.25 on stage 3).

**FIGURE 2:**
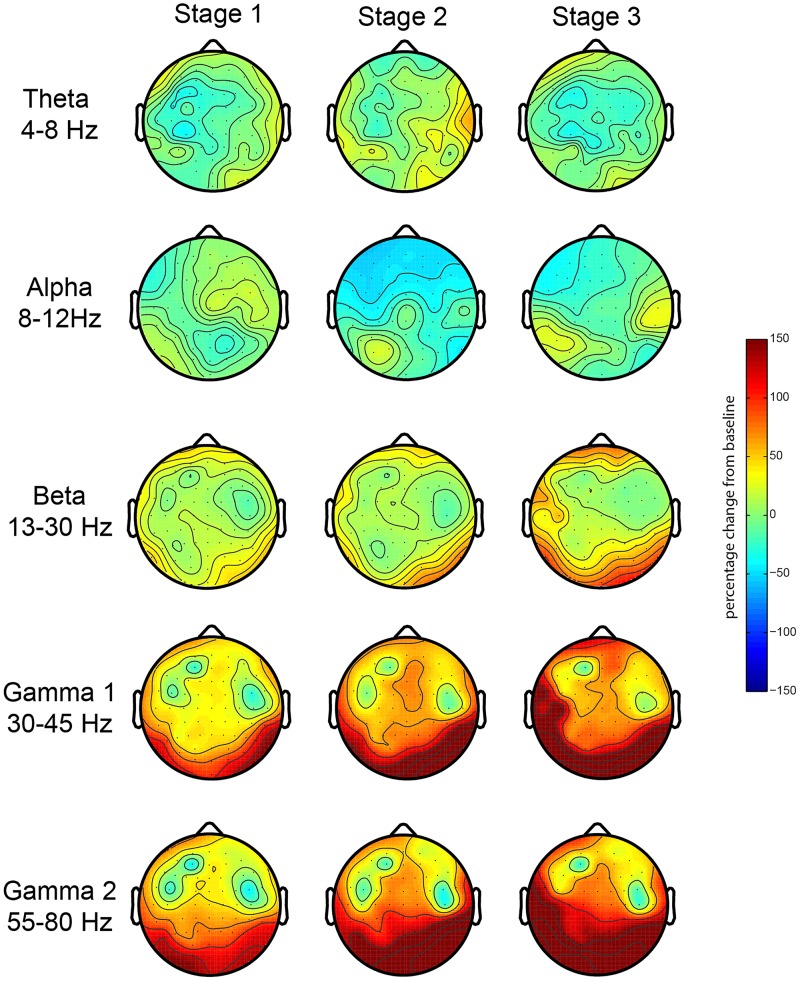
EEG oscillations during the first meditation session. Gamma power changes from baseline during each stage or deepness of meditation (Stages 1, 2, 3) as indicated by the artist in the first session (sessions 1A and 1B). We analyzed the relative power over the traditional frequency bands: theta (4–8 Hz), alpha (8–12 Hz), beta (13–30 Hz), gamma 1 (30–45 Hz), and gamma 2 (55–80 Hz) as the percent signal change from stage 0.

**FIGURE 3:**
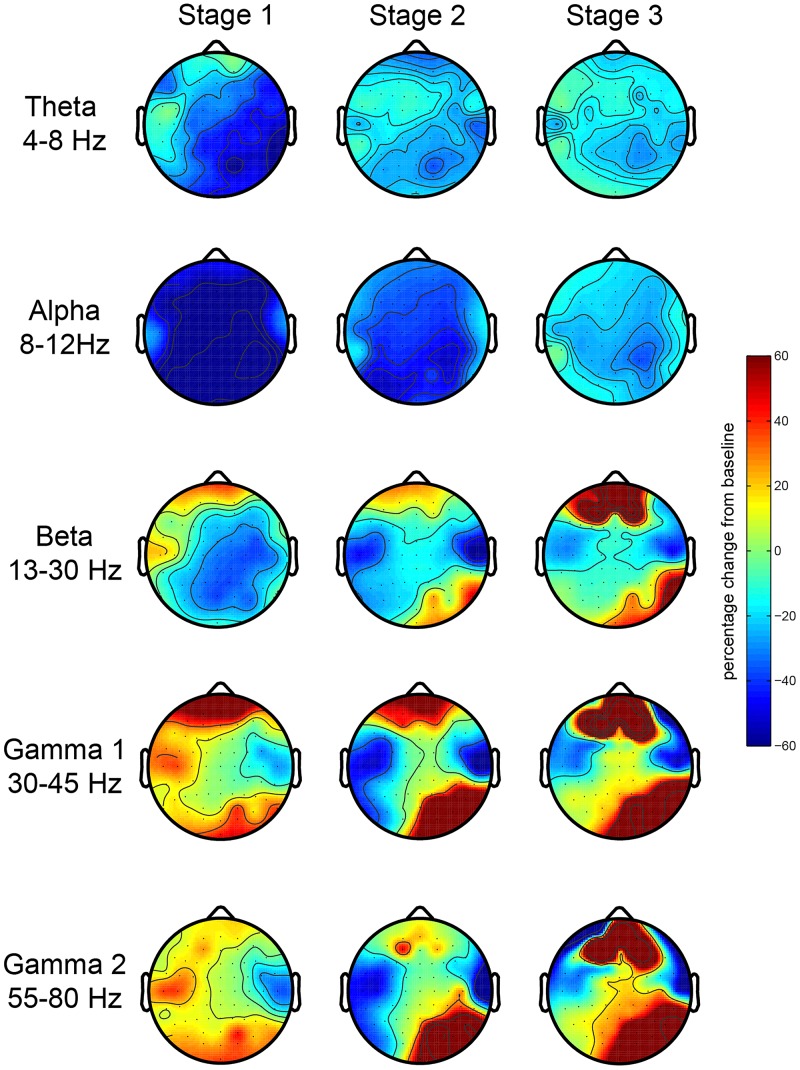
EEG oscillations during the second meditation session. The figure display the gamma power changes from baseline during each stage or deepness of meditation (Stages 1, 2, 3) as indicated by the artist in the second session (sessions 2A and 2B). We analyzed the relative power over the traditional frequency bands: theta (4–8 Hz), alpha (8–12 Hz), beta (13–30 Hz), gamma 1 (30–45 Hz), and gamma 2 (55–80 Hz) as the percent signal change from stage 0.

First, we investigated the EEG oscillatory correlates of the meditation and its various stages. We analyzed the relative spectral power changes in each of the three stages of the meditation (as indicated by L.C.) in relation to baseline, which was at the start of the meditation. The results for the first session showed a robust increase in posterior gamma power (both gamma 1 and 2), which was larger toward stage 3 (Figure 2). There were no robust signal changes in other frequency bands. In order to test the consistency of this finding, we repeated our analysis for the second meditation session, and the results were quite similar, but with an added increase in frontal gamma power (Figure 3).

**FIGURE 4:**
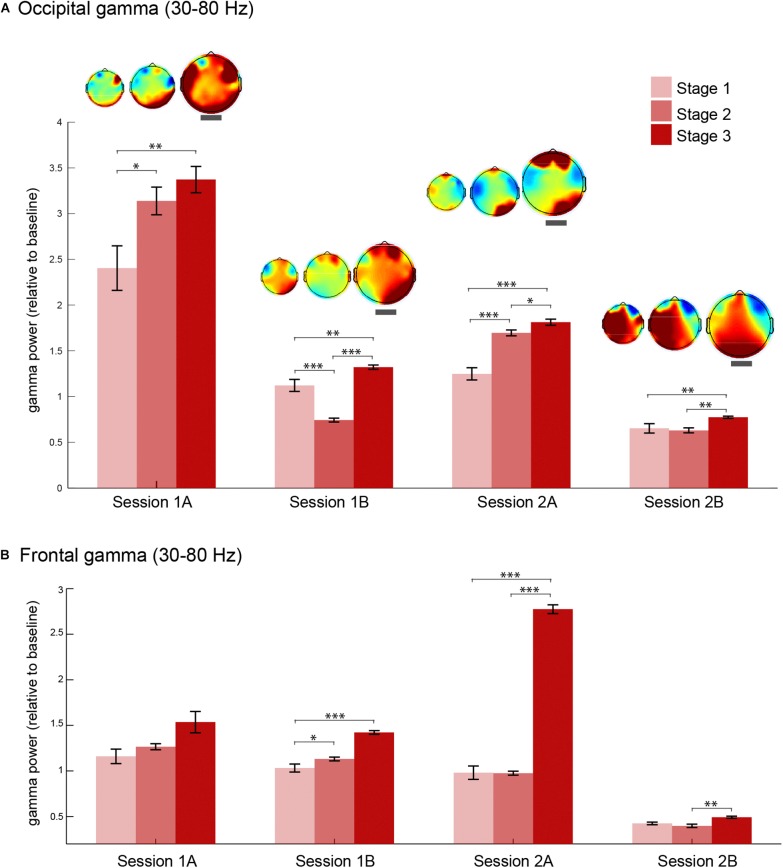
Gamma oscillations during each stage of meditation in four sessions. **(A)** Occipital (O2, Oz, and O1) gamma power (30–80 Hz) during stages 1, 2, and 3 of meditation in the two main sessions (days 1 and 2) and the corresponding meditation sessions (**A** and **B** as first and second meditation round). The topography of gamma power in relation to baseline for each session is presented above each error bar. The topographical maps highlighted with a thick gray line correspond to stage 3 of meditation. **(B)** Frontal (AF4, AFz, and AF3) gamma power (30–80 Hz) during stages 1, 2, and 3 of meditation in the two main sessions (days 1 and 2) and the corresponding meditation sessions (**A** and **B** as first and second meditation round). The error bars represent +/–1 S.E.M. The asterisks represent the pairwise comparisons (Bonferroni Corrected) between the conditions: ^∗^*p* < 0.05, ^∗∗^*p* < 0.01, ^∗∗∗^*p* < 0.001.

Next, we explored the consistency of this increase in gamma oscillations during stage 3 of meditation by analyzing gamma power changes in four meditation sessions. We extracted gamma power from occipital (O2, Oz, and O1) and frontal (AF4, AFz, and AF3) electrodes in each stage of meditation in all sessions (Figure 4). Occipital gamma power increased significantly in the stage 3 of meditation in all sessions regardless of the baseline level (Figure 4A), and associated scalp topographies of gamma power in each stage indicate that gamma oscillations increased especially in the occipital regions independent of how much it changed from the baseline. In addition, the relative change in the occipital area seems to be lower in the second session of the day (sessions B), which could be due to some carry over effects on the baseline coming from the previous meditation session.

Lia Chavez reported experiences of intense visual imagery only during stage 3 of meditation, stage that she denominated as analytical contemplation in which she reported to simply observe her inner visions. Due to our earlier findings on the gamma band, we conducted three other meditation sessions targeting the occurrence of the specific visions in relation to the oscillations. According to L.C., her visions occur as specific events and do not have a clear shape. In her words “*they are abstract, they look like volcanic explosions*”; they occur spontaneously with varying durations.

### Meditation tACS Sessions

In order to understand the nature of these spontaneous creative visions and whether their content could be modulated by brain stimulation, we performed three brain stimulation conditions during the meditation of L.C. as follows: (1) Occipital gamma tACS (40 Hz at PO7 and PO8); (2) Alpha tACS (10 Hz at PO7 and PO8); and (3) Sham (30 s at 40 Hz on PO7 and PO8). These conditions were carried out in separate days and L.C. was blind to the stimulation condition. During each session, the first meditation practice was done simultaneously to the brain stimulation followed by the EEG procedures for a second session monitoring EEG without brain stimulation. During each stimulation condition, L.C. indicated the onset and the offset of each individual vision by pressing two buttons in the response box. We measured the frequency and duration of each vision during three different brain stimulation conditions.

**FIGURE 5:**
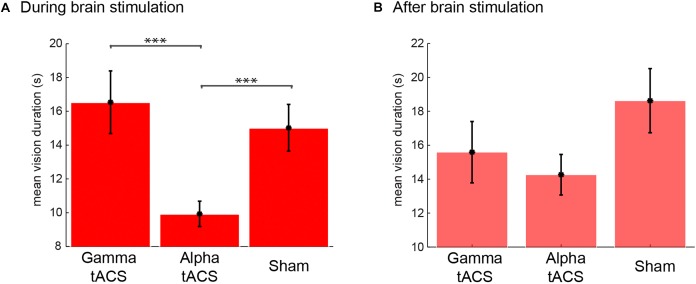
Duration of visual imagery events during and after tACS brain stimulation and sham. Mean vision duration (s) during **(A)** and after **(B)** tACS in gamma and alpha frequency and sham.

After each session, L.C. reported her own experiences during the meditation. For meditation during alpha tACS, she experienced 175, in total, spontaneous visual imagery events. Importantly, if one vision was followed by another, there was no offset button press but another vision button press. The results indicated that her visions were about 15 s long (see Figure 5 for the average duration in each condition). For the meditation following alpha tACS, she reported that the experienced visions were strange and excessively sharp as compared to her usual experiences of vision with low resolution. On a scale from 1 (undefined, low resolution) to 5 (sharp, well defined), she rated the visions as 5 (after the session, the visions in general, not each vision individually). She reported that “*I felt like the images were invading my thoughts, sharp, very sharp images.*” During the gamma tACS, she experienced 106 visions, reporting that “*the images were more like what I usually experience during my meditation*,” and rated the images (after the session was finished) as one (very blurred) on the sharpness scale mentioned previously. During the sham stimulation, she experienced 118 visions and rated them as a two on the referred scale. She reported that the visions were very similar to the ones she usually experiences. In order to statistically compare whether the brain stimulation had any effect on the duration of these visions (Figure 5), we conducted an one-way ANOVA comparing the *vision durations* (each individual vision as a data point, meeting all assumptions for ANOVA) between sham, gamma and alpha tACS and found that the brain stimulation significantly modulated the duration of those visions [*F*_(2,395)_ = 12.39, *p <* 0.001]. *Post hoc* contrasts (*Bonferroni corrected)* showed that the visions were significantly shorter during alpha tACS than gamma (*p* < 0.001) and sham (*p* < 0.001) brain stimulation. There was no difference between gamma and sham stimulation (*p* = 0.958). Therefore, these findings show that alpha tACS targeted at the occipital region can modulate the duration of visual imagery during meditation.

Immediately following the meditation session with brain stimulation, the EEG cap and electrodes were set up and a second meditation session was recorded in each stimulation day. There was a 35-min gap for setting up the EEG cap between the end of the stimulation and the following meditation with EEG. Although there was a trend for lower vision duration in the session following gamma and alpha tACS (Figure 5B), the effect was not statistically significant [*F*_(2,330)_ = 2.14, *p =* 0.120]. However, we observed a larger number of visions following alpha tACS (*n* = 141), and the same number of visions for gamma tACS (*n* = 96) and sham (*n* = 96). *Post hoc* contrasts (*Bonferroni corrected)* between vision durations between conditions revealed no significant differences between any of the conditions in the post stimulation session. Interestingly, after the session following the alpha tACS stimulation, L.C. reported that her visions were more normal than during the stimulation although still sharper than usual (rated as a 3 from 1 to 5). In the sessions following gamma brain stimulation, the subject reported that the visions were similar to what she usually experiences. However, she reported that she was very tired on the session following the sham stimulation (especially due to train delays she faced in the morning). She reported that her meditation was not successful because she was feeling fatigued. She reported that the imagery she experienced during that EEG session was “*not interesting, it was just like unconscious junk, it did not feel like proper imagery and it was sharper than usual.*” Therefore, in the EEG following sham stimulation, the nature of the imagery she reported was very distinct from the usual inspiring visual imagery she experiences during stage 3 of meditation.

### Meditation EEG Following tACS: Occipital Gamma During Visions

**FIGURE 6:**
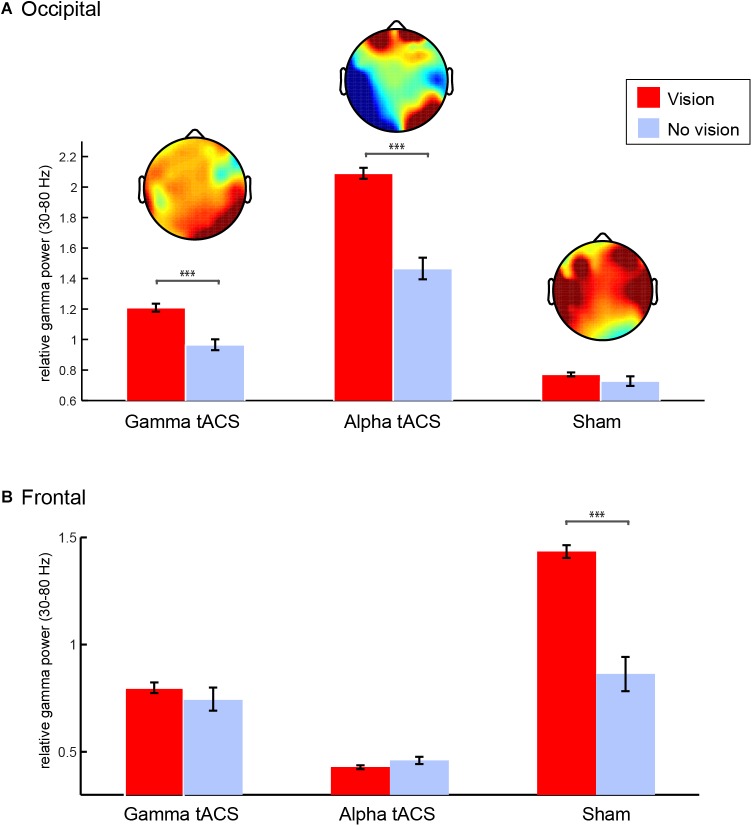
Gamma oscillations during spontaneous visual imagery. **(A)** Right occipital gamma (35–80 Hz) power (P8, P10, PO4, PO8, O2) during visions (red) vs. no visions (blue) during meditation stage 3 following gamma, alpha, and sham tACS conditions. **(B)** Frontal gamma (35–80 Hz) power (F3,F4,T7,T8,C5,C6) during visions (red) vs. no visions (blue) during meditation stage 3 following gamma, alpha, and sham tACS conditions. Error bars represent +/–1 S.E.M across epochs (vision and no vision epochs of 2 s each). ^∗∗∗^*p* < 0.001.

Considering that her technique (see “Methods”) is slightly different in stages 1 and 2 as it includes the usage of stabilizing strategies such as repetition of mantra, there is a possibility that the increase in occipital gamma is a result of a change in activity during stage 3, in which she reports a pure contemplative state. Therefore, we asked whether gamma was indeed related to the intense visual imagery experienced by L.C. In order to understand the visions she experienced during the meditation, we segmented the data of stage 3 into 2 s epochs according to her indication of whether she was or not experiencing a vision. We focused on gamma power over occipital electrodes since this was the main oscillatory correlate of her meditation during stage 3. We observed that right occipital gamma power was higher during visions compared to no visions following gamma and alpha tACS, but not following sham (Figure 6). After sham, gamma power increased over the frontal but not the right occipital electrodes. Interestingly, this was the session that L.C. reported a high level of fatigue and the lowest quality of visual imagery as she described as “unconscious junk.”

Because L.C. was experiencing visions most of the time during stage 3, we limited the number of vision epochs in the analysis by randomly selecting the same number of no vision trials for each condition (following gamma, alpha, and sham tACS). We compared right (P8, P10, PO4, PO8, and O2) occipital gamma power (30–80 Hz) during epochs with vs. without visions in stage 3 after gamma, alpha, and sham stimulation conditions (2 × 3 ANOVA). The three conditions differ in terms of relative gamma power [*F*_(2,322)_ = 106.47, *p <* 0.001], which could be related to the quality of the meditation experience during each day. Importantly, our results showed that gamma power was significantly higher while she was experiencing visual imagery [*effect of visual imagery: F*_(1,322)_ = 29.39, *p <* 0.001], but that interacted with *stimulation condition* [*F*_(2,322)_ = 9.99, *p <* 0.001] as the difference between vision and no vision was significant only following gamma and alpha tACS, but not following sham. Instead, there was an increase in gamma power in the temporal and frontal areas during stage three of sham. We conducted the same analysis using gamma power from fronto-temporal electrodes (F3, F4, T7, T8, C5, C6 – Figure 6B) and observed that there was significant increase in gamma power over the fronto-temporal areas following sham [*effects of condition: F*_(2,322)_ = 54.94, *p <* 0.001], which interacted with visual imagery [*F*_(2,322)_ = 10.84, *p <* 0.001] and it was larger for visions compared to no visions [*F*_(2,322)_ = 16.93, *p <* 0.001]. The significant contrasts (*Bonferroni corrected*) can be observed on Figure 6.

**FIGURE 7:**
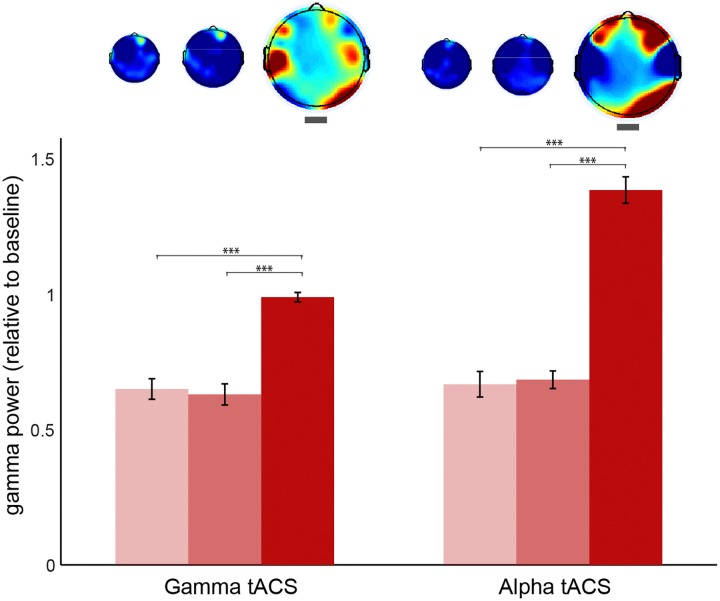
Gamma oscillations during each stage of meditation following gamma and alpha stimulation sessions. Occipital (O2, Oz, and O1) gamma power (30–80 Hz) during stages 1, 2, and 3 of meditation (from brighter to darker red) following gamma and alpha tACS sessions. The power values are relative to baseline (stage 0). The error bars represent +/–1 S.E.M. The asterisks represent the pairwise comparisons (Bonferroni Corrected) between the conditions: ^∗^*p* < 0.05, ^∗∗^*p* < 0.01, ^∗∗∗^*p* < 0.001.

In order to control for other differences in gamma oscillations at stage 3 caused by the simple button press rather than meditation depth and also to test whether the general effects of meditation depth (as in Figure 4) were still present in this session, we compared the stages without separating visions and non-visions in the EEG sessions following alpha and gamma tACS (Figure 7). The results revealed gamma increased in the occipital electrodes in both sessions. The increase was also observed on the left temporal after gamma tACS and over the prefrontal electrodes following alpha tACS. In order to compare the conditions, we conducted a 2 (session: post-gamma vs. post-alpha tACS) × 3 (stage: 1, 2, or 3) ANOVA using the occipital power values (O2, Oz, and O1) as dependent variable. The results confirmed a significant effect for stage [*F*_(2,1223)_ = 5.25, *p* = 0.005] but not for session [*F*_(1,1223)_ = 0.46, *p* = 0.499], nor interaction [*F*_(2,1223)_ = 0.59, *p* = 0.557]. *Post hoc* comparisons revealed that gamma increased during stage 3 more than in stage 1 and 2 (*p* < 0.005, Bonferroni corrected). In order to check whether the increase over the frontal electrodes was also significant we conducted the same factorial ANOVA using gamma power over prefrontal (AF4, AFz, and AF3) as the dependent variable. The results showed no significant effects for session, stage neither interaction between these two (*p* > 0.9), suggesting that this increase was not consistent. We also extracted the power values for the left temporal since we observed an increase during stage 3 after gamma stimulation. We conducted the same factorial ANOVA which revealed no effects for stage, session or interaction (*p* > 0.5), which suggests that the average increase in left temporal gamma was not statistically significant.

## Discussion

Although the spontaneous visual imagery during meditation has been previously reported in the literature as a well-known correlate of meditation (Lo et al., 2003; Lindahl et al., 2013), very little is known about its potential neural correlates. Here we reported a case study of spontaneous visions occurring during deep stages of meditation that are considered as the source of creative inspirations for a reputed professional performing artist. In summary, our study has three main contributions that can help advance our understanding of the interface between creativity, visual imagery, and meditation: (1) we observed that occipital gamma increases in deep stage of meditation and that this increase is built up in the lower stages; (2) we showed that occipital gamma, as observed in the deep stage of meditation, is higher when L.C. experiences spontaneous visual imagery during meditation; (3) for the first time, we demonstrated that it is possible to interfere with visual imagery contents during meditation by delivering tACS to the occipital cortex. Further, by acquiring fine-grained details of the different stages of meditation over repeated sessions, our findings offer a novel insight into the meditation from first person experience, as recommended previously (Thomas and Cohen, 2014).

It is important to notice that in this study we did not test the association between spontaneous visual imagery and the quality of her visual art pieces generated from it. We focused on understanding whether we could consistently identify the oscillatory correlates of her spontaneous visual imagery and whether we could modify it by stimulating specific brain oscillations using transcranial brain stimulation. Nonetheless, the participant reported that very detailed visual imagery, as she experienced during the alpha tACS session, was not very useful for her creative production. According to her, those images were “too detailed” to be used in her artwork which is abstract (see example on Figure 1B). Notwithstanding our limitations as a case study, this might suggest that further studies can potentially look into the association between the contents of spontaneous visual imagery and creativity in visual artists. It has been suggested that the dynamics and the contents of spontaneous thoughts or mind-wandering are important for creativity (Christoff et al., 2016). Our findings seem to suggest that the contents of the spontaneous visual imagery may be important for the creative production in visual arts. As a first case study on this, we suggest that future researchers explore ways of inducing spontaneous visual imagery in artists and investigate its association with creative outputs in visual arts.

Regarding the brain oscillations during meditation, we observed an increase in gamma power (>30 Hz). This increase was higher during stage 3 of meditation and stronger in the occipital areas. Importantly, the subject was able to consistently show a similar pattern (occipital gamma increase) in several meditation sessions. This increase was sometimes accompanied by an increase in prefrontal gamma oscillations, but that was a less consistent pattern across sessions. We did not observe any effects in the lower frequency bands as some studies had found (Berman and Stevens, 2015). However, there are a number of studies which found increases in gamma oscillations during meditation (Lehmann et al., 2001; Lutz et al., 2004; Cahn et al., 2010; Davis and Hayes, 2011; Berkovich-Ohana et al., 2012; Hauswald et al., 2015; Braboszcz et al., 2017) and at rest in experienced meditators (Lutz et al., 2004; Berkovich-Ohana et al., 2012; Thomas and Cohen, 2014). In particular, occipital gamma has been observed during meditation (Lutz et al., 2004; Cahn et al., 2010; Braboszcz et al., 2017). To our knowledge, no study so far has connected the documented increase in occipital gamma during meditation with spontaneous visual imagery. The process of “seeing things” during meditation has been reported as a relatively common phenomenon amongst meditators often reported as encounters with light but has never been investigated using neuroimaging methods (Lo et al., 2003; Lindahl et al., 2013).

We observed that gamma increases were most consistent in stage 3 of meditation, which challenges the idea that gamma represents the general meditation techniques rather than meditative states as suggested recently (Berman and Stevens, 2015). Instead, we suggest that gamma oscillation, in particular occipital gamma, is one of the main mechanisms behind deep meditation states. Importantly, we found this occipital gamma to be associated with creative visual imagery experienced by our subject. When the subject was in deep meditation (stage 3), but was not experiencing these visions, this neural signature was reduced. Interestingly, in day 6 we did not observe such occipital increase in a session that the participant reported the imagery content as “junk” or not proper meditation visual imagery content. One important question is how these visions emerge: does gamma increase because of the visions or a higher gamma triggers spontaneous visual imagery? Our results seem to suggest, by looking at the previous meditation stages, that gamma starts increasing before the visions are experienced, even before the subject reaches a deep meditative state. However, this is only a hypothesis and it requires further investigation and this study has only investigated this process in a single participant, so we can only speculate that heightened occipital gamma may trigger spontaneous visual imagery in experienced meditators, which could explain the well-documented encounters with light experienced by meditators (Lindahl et al., 2013). This phenomenon has not been addressed in the neuroimaging literature up until now and it requires further investigation.

Finally, we demonstrated that by applying tACS to the occipital cortex, bilaterally, it is possible to modulate the content and duration of such visions. Unknown to the participant, alpha tACS seems to have led to unusually sharp visual imagery content (high spatial frequency) with shorter duration, whereas gamma and sham did not modulate vision duration or content. It has previously been shown (Fründ et al., 2007) that occipital alpha increases when processing sharper visual stimuli (>5 cycles per degree – cpd – of visual arc) whereas gamma increases when processing lower resolution images (<5 cpd). Previous studies have shown that it is possible to interfere with visual processing by entraining alpha (Brignani et al., 2013) and gamma (Helfrich et al., 2014; Janik et al., 2015) rhythms in the visual cortex by tACS. In our study, rather than interfering with visual perception, alpha tACS seems to have modified the visual imagery contents during meditation by making them sharper (according to L.C. subject report – blinded to the stimulation condition), which is consistent with the role of alpha oscillations in processing higher spatial frequency visual stimuli (Fründ et al., 2007). This result also evidences that spontaneous visual imagery might rely on similar neural correlates as veridical vision, in the same fashion as the observed shared processes between imagined or learned images and their actual visual processing (Albers et al., 2013; Luft et al., 2015). Considering that in this study we only had a single tACS session for each frequency, these results must be interpreted with caution since there are several factors which could have affect the visual experience of our participant.

On the other hand, gamma tACS did not seem to affect the meditation experience, which was reported by the subject as her usual meditation experience. This finding might have occurred due to the already heightened gamma oscillations in the occipital cortex during meditation since it was found that the tACS effects on the oscillations are highly dependent on endogenous brain states on the stimulated frequency (Neuling et al., 2013). In particular, it was observed that individualized alpha peak (IAF) tACS stimulation only enhanced IAF power under conditions in which the endogenous IAF power was naturally low (Neuling et al., 2013). Therefore, it could be that by stimulating gamma frequency, which is naturally higher during her meditation stage 3, we were not able to enhance them. Further studies could explore the possibility of enhancing gamma oscillations for triggering spontaneous visual imagery in beginners or intermediate meditators since they still have not developed such a self-induced high gamma power increase during meditation. Questions such as whether this would increase the depth of meditation or elicit creative visual imagery are of interest. Another interesting possibility is to induce occipital gamma in order to trigger spontaneous visual imagery for creative purposes in artists.

Some limitations of this study must be kept in mind. First, although the gamma band correlates of stage 3 were replicated in different days/sessions, we did not test the tACS effects in a second experiment. Therefore, the effects of alpha tACS on vision duration and precision should be interpreted with caution. Future studies investigating visual imagery on meditators could test this protocol further in a different order. Second, we cannot rule out differences between visions and non-visions in other frequency bands. In this study, we focused on the gamma band because it was the neural correlate of stage 3 meditation. Other frequency bands could be affected by the visions, but they were not investigated in the present study. Third, we understand that as a single case report, there is a need of more studies investigating the neural correlates of visual imagery and how that can affect the creative process in visual arts. Our study provides preliminary evidence that spontaneous episodes of visual imagery experienced in deep meditation are associated with higher occipital gamma, but new studies with other participants having similar experiences are needed. Importantly, our study raises the possibility of using brain stimulation for interfering with visual imagery contents, a relevant new venue to explore to modulate meditation experience.

## Ethics Statement

This study was carried out in accordance with the recommendations of Goldsmith’s ethics committee with written informed consent from our research participant Lia Chavez. She gave written informed consent in accordance with the Declaration of Helsinki. The protocol was approved by the Goldsmiths ethics committee.

## Author Contributions

CL, JB, and MB designed the research. CL, IZ collected the data. CL analyzed the data. CL, JB, MB, and IZ wrote the manuscript.

## Conflict of Interest Statement

The authors declare that the research was conducted in the absence of any commercial or financial relationships that could be construed as a potential conflict of interest.

## Footnotes

1 http://www.vislab.ucl.ac.uk/cogent.php
